# The health system barriers to a healthy diet in Iran

**DOI:** 10.1371/journal.pone.0278280

**Published:** 2023-01-26

**Authors:** Mohammad Amerzadeh, Amirhossein Takian, Hamed Pouraram, Ali Akbari Sari, Afshin Ostovar

**Affiliations:** 1 Social Determinants of Health Research Center, Research Institute for Prevention of Non-Communicable Diseases, Qazvin university of Medical Sciences, Qazvin, Iran; 2 Department of Health Management, Policy & Economics, School of Public Health, Tehran University of Medical Sciences (TUMS), Tehran, Iran; 3 Department of Global Health and Public Policy, School of Public Health, Tehran University of Medical Sciences (TUMS), Tehran, Iran; 4 Heath Equity Research Center (HERC), Tehran University of Medical Sciences (TUMS), Tehran, Iran; 5 Department of Community Nutrition, School of Nutritional Sciences and Dietetics, Tehran University of Medical Sciences (TUMS), Tehran, Iran; 6 Osteoporosis Research Center, Endocrinology & Metabolism Research Institute, Tehran University of Medical Sciences, Tehran, Iran; Wroclaw University of Environmental and Life Sciences: Uniwersytet Przyrodniczy we Wroclawiu, POLAND

## Abstract

**Background:**

Decreasing calories, salt, sugar and fat intake is considered the golden solution to reduce obesity and cardiovascular diseases (CVDs) related to unhealthy diet. This study aimed to investigate the health system induced barriers to a healthy diet in Iran.

**Methods:**

This is a qualitative health policy analysis. We collected data through 30 semi-structured, face-to-face interviews with purposefully identified experts, including policy-makers, top-level managers and related stakeholders. All interviews were transcribed verbatim, and analyzed with qualitative content analysis, facilitated by MAXQDA 11 software.

**Results:**

We identified six categories of barriers: structural problems within the Ministry of Health and Medical Education (MoHME), i.e. Supreme Council of Health and Food Security (SCHFS); the MoHME’s inadequate stewardship of public health, the short life of the deputy of social affairs within the MoHME and its possible impact on the National Health Assembly; inefficient traffic lights labelling for foods; lack of adequate policies and guidelines for monitoring restaurants and fast foods and insufficient incentive policies for the food industry.

**Conclusion:**

In line with the World Health Organization (WHO) Global Action Plan, in 2015, Iran defined its roadmap for prevention and control of NCDs, i.e. some nutritional interventions. However, different stakeholders including MoHME and other sectors need to provide series of interventions to change people’s approach about food choice so that they might reduce the consumption of foods with excessive salt, fat and sugar.

## Introduction

Non-communicable diseases (NCDs) surpassed 42 million global deaths in 2019 (over 74% of the global deaths) [1]. NCDs, specifically cardiovascular diseases (CVD), cancers, respiratory diseases and diabetes, were the common reasons for 63% of the global Disability-Adjusted Life Years (DALY) in 2019 [2]. Among the chief risk factors, poor diet contributes the most and is higher than tobacco, alcohol and physical inactivity altogether [3]. The high consumption of processed food with high amounts of sugar, salt, and trans fats, combined with the low consumption of healthy foods such as fruit and vegetables, whole grains, seafood, nuts and pulses are the main reasons for this problem worldwide [4]. Further, the overwhelming use of western diet styles, including foods with a high amount of salt, sugar and fat, in many low and middle-income countries (LMICs), might have led to widespread degenerative diseases [5,6].

A study found that 1.65 million annual global deaths attribute to diseases related to elevated salt intake, i.e. CVD and cancer [7]. The saturated fatty acids (SFA) also increase the dyslipoproteinemia risk, which leads to raising LDL cholesterol and probably CVD risk [8]. Excessive intake of SFA also increases the risk of breast cancer [9]. Besides, there is evidence that sugar-sweetened beverages intake contributes to growing overweight/obesity, diabetes type II, and metabolic syndrome [10].

To overcome the problems related to obesity and CVD, the main focus is on promoting healthy diets through decreasing calories, salt, sugar and SFA intake [11]. WHO attributed 82% of annual death to NCDs in Iran (43% cardiovascular diseases, 16% cancers, and 23% other NCDs) in 2020, which significantly burdens the health system. Since dietary risk factors are the leading cause of NCDs in Iran, diverse interventions, i.e., improving unhealthy diets and decreasing salt, sugar, and fat consumption at both industrial and population levels are among the primary health sector interventions to reduce the mortality related to NCDs [12,13]. This study aimed to investigate the health system induced barriers to a healthy diet in Iran.

## Methods

### Study design & data collection

This is a qualitative study. We collected data through 30 semi-structured, face-to-face interviews with purposefully identified experts, including policy-makers, top-level managers and related stakeholders from December 2018 until August 2019 in Tehran- Iran. We developed and used a generic interview guide for data collection via literature review and using the Government Healthy Food Environment Policy Index (Food-EPI) monitoring tool. The guide had two components: policies and infrastructures. We used the health policy triangle framework for deductive analysis [13]. The participants were provided with an information sheet, which described the study aims, asked their consent for participation, and reassured them about data confidentiality and anonymity. We conducted all interviews in the interviewees’ workplace. The interviews were digitally recorded, transcribed verbatim and analyzed.

### Setting and sampling

We used snowball approach for sampling and continued the interviews until we reached saturation, so no new themes were emerged. Table 1 displays the characteristics of the study participants.

**Table 1:** Characteristics of the study participants.

Organization	Sector	Numbers
Ministry of Health and Medical Education (MoHME)	Community Nutrition Office	2
	National Committee for Control and Prevention of NCDs	2
	Supreme Council for Health and Food Security	2
	NCDs’ Directorate	1
	Deputy of Curative Affairs	1
	Non-Communicable Diseases Research Center affiliated with TUMS	1
	WHO Office in Iran	1
	Deputy of Public Health	1
	Primary Health Care (PHC) Network Office	1
	Health Education and Promotion Office	1
	Food and Drug Administration	3
	Iranian Academy of Medical Sciences (AMS)	1
Universities of Medical Sciences	Deputy of Public Health	2
	Schools of Nutrition	2
Ministry of Agriculture	Environment and Food Safety Office	1
Ministry of Industry, Mine and Trade	(Health, Safety and Environment) HSE Department	1
Ministry of Education	Deputy of Health and Physical Education	1
Municipalities	Tehran HSE Department	2
National Standard Organization	The Department of Food Industries	1
Ministry of Economic Affairs and Finance	Department of Health	1
Islamic Republic of Iran Broadcasting (IRIB)	Policy-making Council	1
Planning and Budget Organization	Welfare and Health Affairs Department	1
Total		30

### Data analysis

We carried out the qualitative content analysis using deductive and inductive approaches for data analysis, facilitated by MAXQDA 11 software (VERBI software, Germany, https://www.maxqda.com). We used MAXQDA qualitative data analysis software to facilitate a constant-comparative coding process and identify the themes. According to this approach, the data was analyzed in five stages, i.e., familiarization, identifying a thematic framework, indexing, charting or mapping, and interpretation. After the open coding, we categorized the codes into multiple groups and then compared the codes with each other, changing constantly to represent the message of the interviews. The first and corresponding authors carried out the categorization process and all authors revised and approved the entire steps. In the end, to increase the credibility, we sent the transcripts, categories, subcategories and codes to several interviewees and sought their approval.

### Ethical approval and consent to participate

The Tehran University of Medical Sciences (TUMS) approved this study via the Ethical Committee (the ethical code: IR.TUMS.REC.1397.193). All methods were carried out in accordance with relevant guidelines and regulation. We provided the interviewees with an information sheet, described the purpose of the study for them, and obtained their written consent.

## Results

Our main findings on health-sector determinants of diet are summarized in Table 2. Participants reported different determinants. Some interviewees mentioned structural problems in some of MoHME’s departments, its inadequate stewardship of public health and the short life of the deputy of social affairs within the MoHME. Some problems were inefficient traffic lights labelling for foods and lack of adequate policies and guidelines for monitoring restaurants and fast foods. Insufficient incentive policies for the food industry was the last issue.

**Table 2:** Health-sector determinants of diet in Iran.

Theme	Subtheme	Examples of health factors
Health related factors	Structural problems in some MoHME’s departments	Iranian Non-Communicable Diseases Committee (INCDC)
	The MoHME’s inadequate stewardship of public health	the MoHME does not have enough power to deal with other sectors that endanger public health
	The short life of the deputy of social affairs within the MoHME	its possible impact on the National Health Assembly
	Inefficient traffic lights labelling for foods	it is too small, the volume of the package is small and it is not possible to use larger labelling
	Lack of adequate policies and guidelines for monitoring restaurants and fast foods	There are not enough policies on the type of raw materials, an acceptable amount of using salt, fat and sugar in the dishes
	Insufficient incentive policies for the food industry	More incentive and agreement mechanisms between industries and the MoHME is required

### Structural problems in some MoHME’s departments

One problem related to the healthy diet and reducing the consumption of sugar, salt, and fat consumption is the current structures within the MoHME. One structure is the INCDC, which has some problems. Given that the control and prevention of NCDs is an inter-sector task and requires the other sectors’ cooperation, this committee does not have enough power to draw the necessary cooperation and actions outside the MoHME:

*"The Committee does not have a legal and regulatory structure. For example, the budget line and financial support. It only acts as a coordinator and cannot direct the resources of the various sectors.”(PMN10)*

Another important structure in this field is the Supreme Council of Health and Food Safety (SCHFS), an inter-sector structure. The council also has some problems. For example, other ministries that are members of this council do not cooperate appropriately, and it leads to many implementation problems:

" *The council itself states that a maximum of 20 have successfully implemented out of 100 resolutions that we have submitted to the president for approval*.*" (PMN8)*

Besides, some ministers’ presence in the SCHFS is not permanent, and their absence causes some resolutions are not properly approved and implemented:

*“In the SCHFS, many resolutions are not implemented. At one meeting of the SCHFS, the Vice President complained about the absence of the ministers. There was no minister other than the Minister of Health, and it caused that they did not discuss many issues. The Vice President and the Minister of Health approved many resolutions. They passed thirteen resolutions in this way. “(PMN8)*

Another important structure is the Community Nutrition Office, which takes many actions about a healthy diet in the community. Some interviewees believed that the Community Nutrition Office does not have the necessary executive power and a proper executive structure for community nutrition programs, and most times, its tasks interfere with other sectors:

*“For example, we have the Community Nutrition Office, and its roles are passive because the Deputy for Public Health does not specify a special role for it.” (PMN10)*

### The MoHME’s inadequate stewardship of public health

It seems that MoHME has many problems in terms of stewardship and structure, and it does not have enough stewardship power. When other sectors endanger public health, the MoHME does not have enough power to deal with them. In other words, the Minister of Health cannot prosecute other ministers’ actions against health:

*"There is no accountability about health in other sectors, e.g. industry, agriculture, education…and the MoHME is not in a position to prosecute them. There are many ministers and presidents in the SCHFS, but the health minister is an ordinary minister.” (HEN13)*

### The short life of the deputy of social affairs within the MoHME

One measure in forming NGOs and drawing people’s participation in health was the establishment of the National Assembly of Health, according to which provincial and city assemblies should be formed. These assemblies can become more aware of the public health status in the cities and neighborhoods and cause more people’s participation. Nevertheless, due to the short life of the deputy of social affairs within the MoHME, it will not be easy to form an assembly and continue its work:

*“One problem is that the MoHME has removed deputy social affairs from its structure, and we have no administrative structure to follow up on the National Health Assembly. As long as there is no administrative structure and work is only done in the headquarters, the implementation will be disrupted. (HEN24)*

Another possible consequence of its removal is that our information is insufficient at the neighbourhood level. Many decisions are based on national information, and we cannot make accurate decisions. Nevertheless, many measures in NCDs require accurate information from the community, and provincial and city assemblies can help:

*"We are configuring the community to be able to compare them based on neighbourhood policy. We don’t have information at the neighbourhood level, while it is vital. Our information is in the province level.” (HEN11)*

### Inefficient traffic lights labelling for foods

The Iranian Food and Drug Administration (FDA) has implemented traffic light labelling in the food industry in recent years. Its purpose is to help people make better choices. Nonetheless, it is too small, even though in some foods, the volume of the package is small and it is not possible to use larger labelling:

*"The current labelling isn’t very helpful to the consumer at the moment because they’re too small and illegible. The colours are visible, but the figures can’t be read ". (PMN2)*

### Lack of adequate policies and guidelines for monitoring restaurants and fast foods

According to experts, the MoHME has taken noticeable actions in packaged foods. However, there is a gap in restaurants, fast foods and non-industrial foods in which there are not enough policies on the type of raw materials, an acceptable amount of using salt, fat and sugar in the dishes:

*“In restaurants, there aren’t proper policies. Most policies are related to the food industry. Many actions should be taken, such as the amount of food that needs to be served and the amount of oil and salt used. Policy-making is a continuous process that needs revising most of the time.” (PMN27)*

There seems to be no distinct guidelines, and standards for consuming sugar, salt and fat in restaurants. Another interviewee noted:

*“There have been many measures on salt and awareness about the recommended amount at the table, and it has really changed. In the past, salt was in a bowl that changed to the saltshaker, and now people use less salt on the table. Nevertheless, we don’t have any guidelines for restaurants and fast foods about the recommended amount of salt, sugar, and fat consumption. We don’t have enough standards in this regard. This is an area that needs special attention.” (HEN18)*

Furthermore, one common initiative in the world is that restaurants and caterings include the calorie amount of food and its content in the food menu, which helps people make the right decisions about food choices. This measure has not been taken in our country yet:

“*Restaurants can write the calories*, *salt*, *sugar and fat content of food on the menu*, *and it’s happening in many countries*. *We have done nothing about it*, *and the FDA hasn’t intervened*.*” (PMN26)*

### Insufficient incentive policies for the food industry

Some experts believe that administrative policies, including government and regulatory tools for the food industry, have been mandatory in industries to reduce sugar, fat and sugar. In many countries, producers have reduced the amount of salt, sugar and fat based on incentive and agreement mechanisms between industries and the MoHME:

" *For example*, *the head of the FDA said five or six years ago that I would order all the salts in the country to reduce*. *Nothing happened because it was a wrong order*, *and the mandatory system did not work*.*" (FM4)*

Many experts believe that appropriate educational tools must be used to change society and promote a culture, and actions cannot be taken obligatorily. NCDs are also connected to the lifestyle, and many changes need the participation of the people and society:

“*If a society’s health is threatened in a cultural situation*, *a force cannot change it*. *We should recruit the society and change the beliefs gradually*.*” (HEN6)*

## Discussion

This study aimed to investigate the health system induced barriers to a healthy diet in Iran. This study demonstrates that one problem is related to the structures of some departments within the MoHME, e.g. INCDC does not have enough power to draw cooperation of other stakeholders outside the MoHME. Besides, in the SCHFS, other ministries do not cooperate completely, or some ministers are often absent, leading to implementation problems. Another study also reveals that the SCHFS has some fundamental problems, such as the weak implementation of the proposed strategies, lack of a desirable monitoring and evaluation system, weak intersectoral coordination, and problems related to the peripheral capacities [14]. In general, the MoHME does not have enough executive power in government to deal with sectors that endanger public health. Community Nutrition Office also does not have the necessary executive power and often interferes with other sectors. Another study conducted in Iran recommended setting up a committee of specialists of diverse stakeholders with enough power from different sectors to reduce salt consumption and improve a healthy diet [15]. Besides, another study also highlights that the Nutrition Office structure in Iran needs revising and improving according to its significant tasks and inter-sector and intra-sector relationships need revising [14]. It also seems that with the removal of the deputy social affairs from the MoHME, the continuity of the National Health Assembly and the provincial and city assemblies will face some difficulties. Managing the multi‐sectoral cooperation and enhancing the collaboration between health and non‐health stakeholders is necessary to implement the nutrition policies [14].

Further, one impressive step is using traffic light labelling in the food industry. Other studies in Finland and Brazil have shown that food labelling can be potentially effective [16]. Nonetheless, it has some problems and can be improved for better usage. For example, sometimes it is too small, which needs to be magnified and legible as much as possible. A study conducted in Iran also addresses the small size of traffic lights, and also it is hard to substitute the foods with the red symbol [17]. Another recommendation is to apply digital food activism to solve this problem [18]. Other studies suggest that despite having a good condition in terms of WHO recommendations, the FDA should revise the traffic lights based on the product nature to rank the fat, sugar, and salt content. For instance, the red colour of fat in oil or essential ingredients or green colour in diet cokes might confuse the consumer [14,19].

There are also problems with restaurants, fast foods and non-industrial foods in which there is a lack of policy on the type of raw materials acceptable amount of salt, sugar and fat in dishes. Restaurant food often has more calories, fats, and carbohydrates than homemade foods [20]. A study in Korea found that the sodium amount of foods in restaurants and work cafeterias is more than in homemade foods, and eating out is increasing. It highlights an urgent action to decrease sodium intake via public health strategies at restaurants and other practical measures [21]. It also shows that interventions such as enhancing knowledge about sodium intake in restaurant’s owners and chefs can develop nutritional behaviours and cuisines at restaurants [22].

Another significant problem is that, unlike many countries where industries operate voluntarily, most of the country’s food industry policies have been mandatory. There is a standard top-down system most times, and it is not easy to implement these standards. The other study found that the nutrition labelling plan has a top-down approach in policy formulation and implementation, which might adversely influence the impact of the policy [14]. In high-income countries, programs to reduce salt consumption embrace three strategies: voluntary food reformulation in food industries by assigning targets, applying nutrition labelling, and warning people via education campaigns about the dangers of high salt [23]. The other study recommended a plan containing three phases: In the first phase, the government sets reformulation targets for producers, retailers and restaurants and maximum salt acceptable amount. Meanwhile, campaigns launch to inform the public about the dangers of consuming high-salt foods. In phase two, foods that do not comply with the maximum salt amount must have warning labels. In phase three, The FDA informs consumers about healthy and unhealthy products through mandatory food labelling warning products with high-level salt [24].

### Rigor of study

To the best of our knowledge, this study is the first of its kind to conduct a health policy analysis to identify health-sector determinants of diet in Iran. We were not able to convince a few interviewees to take part in our research despite our utmost efforts, perhaps because they were concerned about their position. However, the in-depth nature of the interviews with different stakeholders enabled us to collect a reliable data source. Besides, we conducted this study before the COVID-19 pandemic. We acknowledge that peoples’ diet might have changed for several reasons, e.g. economic consequences and food accessibility, particularly among vulnerable citizens. Our analysis is contextual-based and applicable to Iranian society characteristics. Caution is necessary to generalize far-reaching conclusions from our study.

## Conclusions

Iran has created a noticeable roadmap in line with WHO recommendations for controlling and prevent NCDs and nutrition programs. However, different stakeholders including MoHME and other sectors need to provide series of interventions to change people’s approach about food choice so that they might reduce the consumption of foods with excessive salt, fat and sugar. In line with sustainable development goal (SDG) 3.4 to reduce 30 percent of premature death due to NCDs and related risk factors by 2030 in Iran, paying more attention to these issues can help the government control NCDs better and reach the target in the long run.

## Supporting information

S1 File This is the S1 interview guide. (DOCX)

S1 Dataset (XLSX)
